# Aboriginal Health Workers experience multilevel barriers to quitting smoking: a qualitative study

**DOI:** 10.1186/1475-9276-11-27

**Published:** 2012-05-23

**Authors:** Anna P Dawson, Margaret Cargo, Harold Stewart, Alwin Chong, Mark Daniel

**Affiliations:** 1 University of South Australia, Sansom Institute for Health Research, Social Epidemiology and Evaluation Research Group, GPO Box 2471, IPC: CEA-01, Adelaide, South Australia, 5001, Australia; 2 Aboriginal Health Council of South Australia, 9 King William Road, Unley, South Australia, 5061, Australia

**Keywords:** Aboriginal people, Australia, Health care professionals, Tobacco and health, Smoking cessation, Qualitative research

## Abstract

**Introduction:**

Long-term measures to reduce tobacco consumption in Australia have had differential effects in the population. The prevalence of smoking in Aboriginal peoples is currently more than double that of the non-Aboriginal population. Aboriginal Health Workers are responsible for providing primary health care to Aboriginal clients including smoking cessation programs. However, Aboriginal Health Workers are frequently smokers themselves, and their smoking undermines the smoking cessation services they deliver to Aboriginal clients. An understanding of the barriers to quitting smoking experienced by Aboriginal Health Workers is needed to design culturally relevant smoking cessation programs. Once smoking is reduced in Aboriginal Health Workers, they may then be able to support Aboriginal clients to quit smoking.

**Methods:**

We undertook a fundamental qualitative description study underpinned by social ecological theory. The research was participatory, and academic researchers worked in partnership with personnel from the local Aboriginal health council. The barriers Aboriginal Health Workers experience in relation to quitting smoking were explored in 34 semi-structured interviews (with 23 Aboriginal Health Workers and 11 other health staff) and 3 focus groups (n = 17 participants) with key informants. Content analysis was performed on transcribed text and interview notes.

**Results:**

Aboriginal Health Workers spoke of burdensome stress and grief which made them unable to prioritise quitting smoking. They lacked knowledge about quitting and access to culturally relevant quitting resources. Interpersonal obstacles included a social pressure to smoke, social exclusion when quitting, and few role models. In many workplaces, smoking was part of organisational culture and there were challenges to implementation of Smokefree policy. Respondents identified inadequate funding of tobacco programs and a lack of Smokefree public spaces as policy level barriers. The normalisation of smoking in Aboriginal society was an overarching challenge to quitting.

**Conclusions:**

Aboriginal Health Workers experience multilevel barriers to quitting smoking that include personal, social, cultural and environmental factors. Multidimensional smoking cessation programs are needed that reduce the stress and burden for Aboriginal Health Workers; provide access to culturally relevant quitting resources; and address the prevailing normalisation of smoking in the family, workplace and community.

## Introduction

Since the emergence of the first case–control studies linking smoking and cancer in 1950 [1-3], government action on tobacco in Australia has become increasingly comprehensive. Measures have included banning of direct radio and television advertising by 1976, and elimination of all print media advertising and sponsorship by the mid 1990’s [4]. Mass media campaigns emerged at the state level in the 1980’s and a comprehensive National Tobacco Campaign was launched in 1997 [5]. Implementation of Smokefree workplace policies began in 1986, and Smokefree policies for enclosed public spaces existed in most states and territories by 2000 [6]. Individual-level smoking cessation interventions also became available, such as nicotine replacement therapy, pharmacological treatments and counseling (face-to-face and telephone). Taxation measures have also made cigarettes increasingly unaffordable: the cost of cigarettes in 2007 included 62.5% in federal taxes [7].

The long-term efforts to reduce tobacco consumption in Australia have been successful in the general Australian population: the last four decades have seen a consistent decline in smoking prevalence from 34% in 1980 to 19% in 2007 [8]. Tobacco control initiatives (e.g. smokefree policies, taxation measures, nicotine replacement therapy, smoking cessation programs, media campaigns) have not been equally effective for Aboriginal and Torres Strait Islander Australians (hereafter referred to as Aboriginal peoples). The first national survey of Aboriginal peoples in 1994 showed that an alarming 55% of the population were current smokers [9]. In 2008, 47% of Aboriginal Australians ≥ 15 years were smokers, more than double the rate of the non-Aboriginal population [10]. DiGiacomo and colleagues [11] posit lack of access and the inappropriateness of existing smoking cessation services for Aboriginal peoples as two factors contributing to the inequitable effect of tobacco control initiatives.

Aboriginal Health Workers (AHWs) are responsible for delivering primary health care to Aboriginal people and are often the first person an Aboriginal client sees when accessing health services [12]. AHWs are employed in both community-controlled Aboriginal health services (which are governed by a board of local Aboriginal community members) and mainstream health services that have a designated Aboriginal Health unit. AHWs have a mandate to coordinate a variety of health programs including smoking cessation advice and support [13]. However, personal tobacco use in AHWs compromises their provision of smoking cessation advice to Aboriginal clients [14-18]. Some AHWs who smoke report that they do not talk to clients about smoking for fear of feeling hypocritical [14]. The continuing high prevalence of smoking in the Aboriginal population may therefore be perpetuated by inadequate support for quitting due to a large proportion of AHWs who smoke: rates of 46-64% in AHWs across Australia are reported [14,18-21]. Prevention of smoking in AHWs must be prioritised because tobacco is the leading contributor to the burden of disease in Aboriginal Australians [22].

Smoking in the Aboriginal population is known to be influenced and perpetuated by a complex range of social, cultural and environmental factors [23-25]. The high prevalence of smoking in AHWs suggests that current tobacco control initiatives inadequately address the needs of this worker population. AHWs are members of the Aboriginal communities they serve and are likely to experience many challenges to quitting smoking that other community members’ experience (e.g. stress, nicotine addiction, social pressures). However, it is plausible that there are also unique contextual factors that make quitting smoking difficult for AHWs and that these factors need to be taken into account in designing smoking cessation programs. Hence, a comprehensive understanding of the barriers to smoking cessation in AHWs was needed to develop effective interventions that reduce smoking in this workforce. We present in this article the findings from an in-depth exploratory qualitative study that aimed to identify the direct and indirect barriers to quitting smoking experienced by AHWs.

## Methods

### Study context, design and theoretical orientation

We undertook this study within a three-year (2009–2011) mixed-method participatory research project investigating smoking in AHWs in South Australia. The ultimate aim of the project was to develop culturally relevant strategies to promote smoking cessation in AHWs. Characterization of the barriers to cessation in this sub-study enabled subsequent identification of the supports AHWs need to quit smoking, and thus was key to achieving overall project objectives. The project was underpinned by the six Iga Warta principles for Aboriginal health projects: a pro-active preventive approach, coordinated activities across sectors, sustainability, consideration of the social determinants of health, and sensitivity to Aboriginal notions of time and space and Aboriginal community and family [26]. University researchers (who were non-Aboriginal) and key members of the Aboriginal Health Council of South Australia (AHCSA) shared decision making throughout the project. Ongoing support was provided by the State-wide Puyu Wiya (No Smoking) Advisory Group (the peak body for decision making related to Aboriginal tobacco prevention and control in South Australia) and the Aboriginal Primary Health Care Workers Forum (the collective body of AHW representatives in South Australia). Mutual trust and respect among partners was developed to promote two-way learning, empowerment, shared ownership and to ensure that culture was respected [27].

We utilised a fundamental qualitative descriptive design [28] to provide a comprehensive, low-inference description of the barriers to quitting smoking. Fundamental qualitative description stays close to the data (‘data-near’) unlike more interpretive qualitative methodologies (e.g. grounded theory, phenomenology) that re-present events in other terms [29]. This straight description of phenomena was selected to provide meaningful data for immediate application in program planning activities. The social ecological paradigm is consistent with Aboriginal notions of health and wellbeing [30], hence we used social ecological theory to inform the study design [31]. Quitting barriers were framed according to multiple levels of behavioural influence: individual factors, interpersonal processes, workplace factors, community factors and public policy [32]. Broader contextual and historical influences that are salient determinants of chronic disease in Indigenous populations were also considered [33].

### Participants and data collection

The State-wide Tackling Tobacco Coordinator, who was a respected elder employed at AHCSA, provided cultural mentorship to the non-Aboriginal members of our research team. Fifteen field visits to metropolitan, rural and remote health services were undertaken in conjunction with the Tackling Tobacco Coordinator where possible, to promote the credibility of our research team and the project. We presented information about the research project and engaged in informal discussions about smoking before inviting local health staff to participate in semi-structured interviews and focus groups. Data collection was sequential in that interviews were conducted first (n = 22), followed by focus groups to clarify emergent findings from interviews (n = 3) and the remainder of interviews (n = 12). The final three of n = 34 interviews yielded no new information indicative of data saturation.

Participants were purposively invited to interviews based on their smoking status (i.e. never smoked, ex-smoker or current smoker) or role within the health service. While the views of AHWs were of greatest interest, other health service staff and personnel working in tobacco control were also invited to participate to capture a broad range of perspectives. In this way, we captured second-hand observations of the challenges AHWs face in relation to quitting, known as ‘shadowed data’ [34], in addition to the direct experiences of AHWs. Thirty-four interviews were conducted in the workplace between August 2009 and August 2010. Interview duration (mean 51 [range 16–133] minutes) was dependent upon availability of time and readiness to share information. Table 1 describes the 23 AHWs, 9 other health service staff and two tobacco control personnel that participated.

**Table 1:** Interview participant characteristics

	**AHWs**	**Health Service Personnel (Managers, Supervisors, Nursing staff)**	**Tobacco Control Personnel**	**All**
Participants	23	9	2	34
Gender (M/F)	10/13	3/6	2/0	15/19
Location				
Metropolitan	9	2	1	12
Rural	13	6	1	20
Remote	1	1	0	2
Ethnicity				
Aboriginal	23	3	2	28
Other	0	6	0	6
Smoking History				
Never Smoked	1	5	0	6
Ex-smoker	9	0	2	11
Current smoker	13	4	0	17

The interview schedule was semi-structured and enquired into the barriers to quitting smoking via a number of avenues. Smokers were asked about previous quit attempts (e.g. *Have you ever tried quitting before?*) and the factors relating to relapse (e.g. *Why did you start again?*). Smokers not wanting to quit were asked why (e.g. *What is stopping you from trying to quit smoking?*), and those currently undertaking a quit attempt were asked about the challenges involved (e.g. *What are you finding the hardest?*). Ex-smokers were invited to tell the story of quitting including what features made it difficult. In this way, challenges to quitting faced by current smokers as well as those recalled by ex-smokers were included in analysis. Non-smokers and management staff were asked about the support AHWs need (e.g. *What do you think AHWs need to help them quit smoking?*) to explore their perceptions of the challenges to quitting faced by AHWs.

Focus group discussions were used to clarify emergent interview findings and were conducted in April and May 2010 following completion of the first 22 interviews. Barriers to cessation identified during interviews that required clarification (i.e. stress, pressure to quit, the role of smoking in social connectivity) were presented to the group to initiate discussion and elucidation. The dynamic of collective discussions was particularly useful in exploring the barriers to quitting that existed at an aggregate (e.g. organisational, cultural) level. Questions were asked around organisational culture (e.g. *Is there a smoking culture in this organisation?*) and the family (e.g. *Is having someone at home who smokes a problem for health workers who want to give up smoking?*). Participation by AHWs as well as a range of other health service staff was welcomed. In total, 17 health service staff (including 4 AHWs, 3 home and community care workers, 2 trainee enrolled nurses and 8 other health service staff) who were 53% female participated in three focus groups (mean 57 [range 45–71] minutes) conducted in regional locations in April and May 2010.

Interviewers were non-Aboriginal and non-smokers, and had previous experience working in Aboriginal health research and in conducting interviews and group discussions. All participants provided written informed consent after being provided with information about the nature of the research. Digital recordings of interviews and focus groups were captured in most instances, and were transcribed verbatim by an external provider. There were three instances where notes were used to record the responses of participants: two participants elected not to be recorded, and one interview with a rural participant was conducted over the telephone. Ethical approval for the research was granted by two institutional human research ethics committees and the South Australian Aboriginal Health Research and Ethics Committee.

### Data analysis

Digital recordings of interviews and focus groups were listened to in full prior to analysis to ensure the analyst was cognizant of the full emotive content of the data (e.g. anger, frustration, sadness). A brief narrative summary was generated for each interviewee. Content analysis of transcribed text and interview notes was based on the method of Graneheim and Lundman [35] and undertaken in NVivo 8 software (QSR International Pty Ltd, 2008). The text was divided into meaning units that included words, sentences or paragraphs related in content and context. Categories were then created that represented internally homogeneous and externally heterogeneous [36] groups of meaning units that expressed the manifest content of the data. Underlying themes that emerged across the categories of quitting barriers were also identified. Themes are “a thread of an underlying meaning through condensed meaning units, codes or categories, on an interpretative level” (p.107) [35]. The analysis was performed in an iterative manner. First AD reviewed all data and identified emerging categories and themes. MC then undertook a secondary review of the subdivision of data and a series of discussions were held to refine the categories and themes. The active engagement of Aboriginal partners and stakeholders in the data interpretation process was considered paramount to the participatory process. A collective member checking process was undertaken (August, 2010) prior to the last round of n = 8 interviews to confirm emerging findings and identify omissions. Findings were presented to and discussed with ten employees (including 5 AHWs) of a metropolitan health service, four of whom also participated in interviews. The barriers to cessation were refined as a result of the stakeholder feedback (e.g. nicotine addiction, not previously elaborated as a barrier to quitting, was described). Two Aboriginal partners (AC, HS) who were members of the research team also reviewed and refined the categories and themes in an ongoing manner to ensure the cultural relevance and rigour of the findings.

## Results

The barriers to cessation presented here were drawn from interviews, focus groups and the collective member checking process. A majority of AHWs wanted to quit smoking and recognised that smoking was bad for their health, negatively affected their families and undermined their role model capacity in the community. Despite their desire to quit, many AHWs hadn’t attempted to quit smoking. Others had made attempts but were unsuccessful. A range of factors related to the individual (beliefs, priorities, knowledge and so on) and pertaining to interpersonal relationships, the workplace, the community and public policy were identified as barriers to quitting smoking in AHWs. It was evident that participants perceived that the social and environmental contexts where AHWs worked and lived created challenges to quitting. Individual AHWs experienced a unique range of barriers that appeared to be dependent upon their personal characteristics, relationships and environmental context. In the text that follows, the barriers to quitting are highlighted in **bold*.***

### Individual barriers

**Stress** and **Grief and Loss** were frequently reported by AHWs and emerged as primary obstacles to quitting smoking and avoiding relapse. AHWs reported a high level of stress due to a multitude of challenges such as health concerns, excessive work demands, inequity in the workplace, family issues, institutionalised racism and the pervasiveness of social disadvantage. Stories of the burden of chronic disease in the families of AHWs and premature deaths in the community were common. The associated grief and loss (particularly premature loss of parents) was associated with increasing the number of cigarettes smoked (in current smokers), or a relapse of smoking (in those that had quit). Experiences of stress inhibited thinking about quitting smoking, as described in this account:

"If there’s a stress, a death or anything else happening in my life you know, I don’t think about giving up, that would be the last thing that’s on my mind at that time. [AHW, smoker]"

A range of **Fears** inhibited some AHWs from embarking on a quit attempt, and being successful in quitting smoking. The concerns included fear of failure (“it’s a shame job”), fear of feeling sick during the quit attempt, fear regarding weight gain, and also fear of losing an effective coping mechanism. As an ex-smoker observed:

"People have this fear of, if I give up, I’m not going to be able to cope with my life; the cigarette, I need the cigarette. [Non-AHW, ex-smoker (shadowed data)]"

There were some, albeit few, AHWs who believed that their **Smoking wasn’t a problem**. Though most AHWs were aware of the risks of tobacco use, respondent’s stories also revealed an attitude (in relation to cancer and other diseases) that “it’s not going to happen to me”. There were AHWs who attempted to rationalise and legitimise their smoking by contrasting their habit with others whose lives were more seriously impacted by tobacco.

"No I don’t see my smoking as a problem because I can go without, I’m not pulling my hair out if I don’t have any, or I’m not spending my last money on it or I’m not going without something just to buy a packet of smokes. [AHW, smoker]"

On the other hand, there were AHWs who knew their smoking was a problem, but shared that **Quitting was not the greatest priority** in their lives. AHWs delayed quitting smoking because they were addressing other health concerns (such as losing weight or controlling their diabetes) or managing housing, family or financial concerns that demanded immediate attention. As a manager observed in relation to AHW staff:

"People have got so many issues, smoking is the least of them, there's just so much who cares, who cares if you smoke there's all this other stuff going on. [Manager, non-smoker (shadowed data)]"

In some AHWs there was a **Lack of knowledge about quit methods.** One respondent suggested that this was a result of doctors having insufficient time to provide adequate smoking cessation advice. Others suggested that once a particular method had been attempted unsuccessfully they were unwilling to try it again, highlighting a misunderstanding regarding the importance of multiple attempts. A range of respondents told of uncertainty about the relative benefit and harms of using pharmacological quit supports. In this account, an AHW smoker spoke of potential harms resulting from ‘patches’ (i.e. nicotine replacement therapy):

"You know there was one old man in town here and he used to smoke all his life and I think young people are very superstitious too, well I am, as a health worker I shouldn’t be like that but yeah, he was smoking all his life and then he put a patch on and I think he had a heart attack and he ended up passing away. [AHW, smoker]"

For those AHWs with an interest in quitting smoking, a **Perceived lack of access to relevant quit tools** was another barrier to quitting smoking. Many respondents felt that the Quitline (a telephone counselling quit support service) was not culturally appropriate for Aboriginal people. One respondent identified that challenges to accessing information on the internet was an obstacle, and others identified the cost of quit supports (e.g. nicotine replacement therapy) created a barrier to quit attempts.

"I’ve even thought about buying patches but when I look at the price of patches and look at the price of cigarettes I say no, cigarettes are cheaper. [AHW, smoker]."

Only a small number of respondents identified that **Nicotine addiction** was a hindrance to quitting. Other personal, social and environmental features were commonly and consistently identified, but biological dependence on nicotine as a challenge to quitting was rarely articulated.

### Interpersonal barriers

While some AHWs shared positive stories about being encouraged to quit, many respondents spoke about a **Social pressure to smoke**. Living with smokers and being surrounded by smokers in social networks contributed to this. A pressure to smoke was often described in social situations where there were a high proportion of smokers present, or when the gathering involved drinking alcohol.

"In that social situation where you're having a few drinks, “come on have a smoke, come on, come on, you smoke” and you just give in because it’s too hard to keep saying no, no, no and you just lose your motivation. [Manager, non-smoker (shadowed data)]"

Many respondents were reticent to give up smoking because they did not want to feel disconnected from their social network or miss out on the social time of the day when smokers congregated together outside. **Social exclusion when quitting** occurred when the family, colleagues or social network of the AHW were offended by their non-participation in smoking. One respondent told of how he regularly provided cigarettes to members of his extended family, and how his family reacted negatively when he decided to quit. His experience of exclusion was so strong that he took up smoking again because sharing cigarettes was central to his role in the family and important for maintaining connectedness. The extent of exclusion experienced by quitters in the workplace is aptly described in this account:

"I mean staff aren't probably as supportive for you quitting as they should be and you sometimes feel like a leper within your own organisation. [AHW, smoker]"

A **Lack of role models and support** also impeded quitting in some AHWs. Many participants knew few friends, colleagues or family members who had successfully quit, and some participants couldn’t identify any successful quitters. A lack of role models engendered the sense that quitting was foreign.

"Interviewer (I): How many people do you know who have successfully quit smoking?"

"Participant (P): You know that’s a good question, off the top of my head, I can’t think of one."

"I: Do you think that might make a difference if you had positive role models around you who had actually succeeded?"

"P: Definitely. [AHW, smoker]"

Furthermore, while some AHWs were supported in their quit attempts, others spoke of a lack of support for quitting in their family and social network, often because people around them were intolerant of mood changes that resulted from the challenges of quitting.

"Well the minute he noticed a slight difference in my moods he’d say ‘You're hanging out for a smoke, why don't you go and buy yourself a packet’ so it wasn't helpful. [AHW, ex-smoker]"

Some smokers experienced **Pressure to quit** from non-smokers which made them feel they were getting ‘picked on’. This experience made some AHWs want to smoke more, and demonstrates that there’s a fine line in the encouragement that AHWs need to move towards quitting.

"[It] became a defiant stand for me because I'm not a person to sort of bend over, or fall down, and so I dug my heels in. [AHW, ex-smoker]"

In a similar vein, a small number of respondents felt that **Unwanted attention during quit attempts** made them more likely to relapse. It seems that even well-wishing comments like “you’re doing such a good job” were a barrier to successfully quitting because they reminded the AHW about their craving for a cigarette.

"I mean if you are constantly reminded of the fact that you smoke or you used to smoke… you think “oh my god, like, I need a smoke” … don't talk about it because now I feel like it. [AHW, ex-smoker]"

### Workplace barriers

Where **Smoking was common in health workers**, quitting appeared to be more difficult. A high prevalence of smoking in Aboriginal health staff perpetuated the sense that smoking was commonplace and therefore acceptable. It also increased the likelihood of being encouraged to smoke by co-workers. In contrast, where there were few smokers in the workplace, a lack of solidarity created a challenge to continuing smoking for this health worker:

"Who have I got to smoke with? It's a very lonely road by yourself. [AHW, smoker]"

Some respondents believed that **Organisational culture enabled smoking**. That is, in some workplaces it was considered acceptable to go outside to smoke throughout the working day without any restrictions placed on smoke breaks. Though uncommon, there were AHWs who noted that smoking with Aboriginal clients was an efficient means to create a common bond.

"If you go and sit on a thing under the tree and have a smoke with them you're going to get a whole bigger story than what you do sitting in the clinic room writing notes. [Manager, smoker (shadowed data)]"

Furthermore, respondents described collective social interactions where staff (smokers and non-smokers) would congregate outside to yarn with a cigarette or cup of tea or coffee. These features of organisational culture were more likely to be present where a high proportion of staff were smokers or when senior managers were smokers. As one participant identified:

"Organisational culture is key to organisational behaviour. If the organisation allows smoking and ongoing smokos then health workers will continue believing that smoking is normal and will be less likely to move towards quitting. [Non-AHW, ex-smoker]"

In some work environments, **Challenges to implementation and enforcement of Smokefree policy** made quitting smoking difficult. In health services with a Smokefree policy, some AHWs admitting getting the urge and having to “sneak” out the front to have a cigarette. Some managers reported that they did not enforce Smokefree policies because it was detrimental to their relationships with staff. Other managers attempted to implement policies but spoke of difficulties in achieving staff compliance.

"I've asked staff members to comply with these policies and procedures, and these dates, but when I look, I still see six people smoking out there. [Manager, non-smoker]"

Implementation of Smokefree policy was particularly difficult where managers were smokers:

"There's some [staff] that are consistently smoking and that might be with, you know, one of their clients or whatever which we've talked about, like, at a team level and said you know we shouldn't be endorsing that. If you're going to have a smoke you need to do it privately. But again, enforcing this is really hard when I'm a smoker. [Manager, smoker]"

### Community barriers

The stories shared reflected that **Smoking was pervasive and acceptable** in many Aboriginal communities. This undermined the motivation for quitting in some AHWs. The acceptability of smoking was perpetuated by significant numbers of Aboriginal smokers and the scarcity of successful quitters. The high prevalence of smoking in community members was believed to be influenced by historical contexts such as the pivotal role of bush tobacco in traditional Aboriginal society and the promotion of manufactured tobacco during and following colonisation. Within this smoking culture, smoking behaviours weren’t questioned or challenged and respondents experienced an **Inability to avoid smoking** in community environments.

"Well I mean I can only say it’s just so hard to get over that addiction when you’re surrounded by so many other people that smoke and it is part of your social time of the day to meet and smoke, yeah it’s terribly difficult. [Manager, non-smoker (shadowed data)]"

While the pervasiveness of smoking in community members made quitting difficult for AHWs, there was a growing awareness of the harms of smoking due to health promotion campaigns and Smokefree policies. One AHW told of hiding his cigarette when out in public so that no-one could see what he was doing, and another health service employee spoke about educating her grandchildren so that the culture of smoking did not continue into the next generation.

"We’re teaching them to say "yucky smoke". [Non-AHW, smoker]"

### Policy barriers

Policy level features were infrequently identified as barriers to smoking cessation in AHWs. There was one respondent, however, who felt that a **Lack of policies to promote Smokefree environments** was a barrier to quitting because smokers were regularly exposed to smoking in the community (such as when going to sporting events, playgrounds, the supermarket, and so on). Another respondent suggested that **Short-term funding of tobacco programs** was an obstacle to quitting because insufficient investment resulted in inadequate support for AHWs in relation to quitting.

"I just hope the funding comes through and that it can be supported for an ongoing period of time, not 1 or 2 years and starts and stop, because we need something sustainable. [Manager, non-smoker]"

### Thematic summary

There were three overarching themes evident in the data. First, though smoking was known to be increasingly prohibited in society (e.g. due to Smokefree policies in restaurants and the workplace) and was restricted in many families (such as not smoking in the home), there remained an overall **normalisation of smoking in Aboriginal society**. There were some AHWs who had anti-smoking influences at home or at work, but a widespread culture of smoking in the extended families and social networks of AHWs perpetuated the notion that smoking was tolerable. Second, **AHWs were burdened** by stress and grief because of multiple responsibilities in their families and workplaces and a multitude of social and environmental stressors (e.g. deaths in the community, dispossession, racism and social disadvantage) which meant that quitting smoking could not be prioritised in their lives. Third, participants spoke of a **scarcity of knowledge, resources and supports for quitting** that were culturally relevant.

## Discussion

This is the first in-depth qualitative exploration of the barriers to quitting smoking experienced by AHWs. Addressing known barriers to quitting smoking is considered a key feature of successful smoking cessation programs in Indigenous populations [11]. Promotion of the health of the Aboriginal health workforce, including prioritising smoking cessation, is considered key to achieving the goals of the South Australian government’s health care plan for the Aboriginal population [37]. Our findings elaborate the range of quitting barriers that exist for AHWs and can be used to inform the development of programs to support AHWs to quit smoking. A reduction of smoking in AHWs could result in improved smoking cessation services and thereafter reduced rates of smoking in the Aboriginal population. This population level benefit is in addition, of course, to potential health gains for AHWs themselves.

Inequitable and challenging environmental conditions provided frequent sources of stress that created a barrier to quitting smoking in AHWs. Stress as a result of racism (in the forms of name calling, denial of cultural identity and discrimination) was frequently reported by AHWs. Systematic review evidence demonstrates self-reported racism is consistently associated with smoking tobacco [38]. In the workplace, AHWs reported stress due to excessive work demands and inequity in employment and training opportunities. These stories mirror the reflections of Mitchell and Hussey [13] who describe that AHWs are "asked to take on many roles at once” and be “everything to everyone” (p.529). The disparity in employment and training opportunities between Aboriginal and non-Aboriginal health staff has been recognised previously [39]. In the extended family, grief and loss due to premature deaths of loved ones, and stress due to the presence of social marginalisation and disadvantage (e.g. poverty, unemployment, housing issues, violence, and drug and alcohol abuse) was commonly expressed. The reduced life expectancy of the Aboriginal population [40] and the association between social economic position and smoking status is demonstrated in empirical data [41]. Our findings indicate that efforts to reduce smoking in AHWs must target environmental stressors that pervade the lives of AHWs and perpetuate smoking.

Culture is highly influential in tobacco uptake, use and cessation [42] and is reflected in lifestyles, ways of living together, values, traditions and beliefs [43]. Nicotine ingestion has a long history in Aboriginal culture. In traditional (pre-colonial) Aboriginal society, nicotine-containing plants (known as *bush tobacco* or *pituri*) were selectively used in social interactions and as a bartering commodity [44]. With the advent of colonisation, Aboriginal peoples were widely exposed to introduced tobacco through rations and wage payments and rapidly became addicted to the physiological effects of nicotine [45]. As a result, manufactured tobacco has become a prominent feature in Aboriginal community life [46]. We found a normalisation of smoking permeated the stories about the challenges of quitting for AHWs. Non-participation in smoking resulted in social exclusion for AHWs and this emerged as a dominant barrier to quitting and a reason for relapse. Communal smoking (i.e., yarning over a smoke) is a longstanding enabler of social cohesion in Aboriginal societies [46,47], and “can be seen as a substitute for traditional activities” (p.6) [47]. Hence, non-participation may be interpreted as a threatening behaviour because it represents a rejection of Aboriginal traditions.

Participants in this study detailed how the act of sharing tobacco was important to promote connectedness with family, peers and colleagues. This reflects the significant kinship obligations of Aboriginal peoples [48]. Sharing of cigarettes as an obligation of the kinship system – that governs social relationships, social control and behaviour – has been observed in other studies of Australian Aboriginal peoples [23,47]. Interpreted from a socio-historical perspective, it could be that the chronology of disrespect and dispossession since white settlement, and the ongoing traumatisation due to racism (institutional and interpersonal) and social disadvantage may propel Aboriginal people towards seeking connection and belonging through shared smoking. In this context, the known deleterious impact of smoking on health is insufficient to motivate smoking cessation attempts in AHWs given the negative effects that would result from the loss of a highly effective socialising tool. An understanding of the barriers to quitting, and future efforts to promote smoking cessation in AHWs, clearly require an appreciation of the cultural relevance of smoking in Aboriginal society. Efforts to promote smoking cessation in AHWs must respectfully challenge the normalisation and acceptability of smoking in the family, workplace and the broader community.

This work extends previous summative studies with Aboriginal health staff where identification of barriers to quitting was a peripheral line of inquiry [20] and included a single focus group discussion [14]. Many of the barriers to cessation experienced by AHWs mirror those experienced by Aboriginal people in general. Social pressure and peer influences [23,49] and a lack of support for quitting [14,47] have likewise been identified as barriers to quitting in other Aboriginal samples. The predominance of certain barriers seems to differ, however, between Aboriginal populations. For example, addiction to nicotine, commonly reported in other Indigenous samples [14,23,24,47], was infrequently identified as a barrier for AHWs. This possibly reflects the unconscious nature of nicotine addiction or that AHWs prefer not to attribute their quitting challenges to a biological addiction. Secondly, stress was a predominant barrier for many AHWs in our study, as in other studies of AHWs [14,20] and community members [50], whereas stress plays a less significant role in other Aboriginal communities [23]. Smoking is associated with low mastery, negative affect and depression in Canadian Aboriginal peoples [51] and it is possible that these features contribute to the stress reported by AHWs. We propose that stress is significant for AHWs because of the challenging nature of their role. Programs to promote smoking cessation in AHWS must address their unique stressors.

AHWs are mandated to provide quality care to clients with complex health and social problems, and must reconcile cultural obligations with the sometimes conflicting expectations of a western health system. In addition to these challenging work demands, AHWs are perpetually grieving the often premature loss of their clients and other community members, and many in government health services report experiences of institutionalised racism [52]. The de-motivating influence of the normalisation of smoking is potentially magnified in those AHWs exposed to pro-smoking organisational culture in addition to widespread smoking in their family and community. Hence, efforts to promote smoking cessation in AHWs must rely not only on individual-level strategies but also include a multidimensional suite of strategies that target the organisational environment. An organisational change process is needed to address workplace stressors and other smoking cessation barriers to create enabling conditions for quitting smoking.

The multi-level and inter-related barriers characterised within the social ecological framework offer clear direction for action planning activities. The identified barriers provide discrete targets for ecologically designed programs. Findings highlight that individual-level strategies are important (e.g. optimised smoking cessation training to address lack of knowledge about quit methods, subsidised nicotine replacement therapy to address nicotine addiction) but will only be effective if implemented alongside community-level strategies (e.g. culturally-relevant social marketing to challenge the normalisation of smoking) and an organisational change process that ameliorates interpersonal-level stressors (e.g. social exclusion when quitting) and workplace-level stressors (e.g. excessive work demands) to create environmental conditions conducive to quitting. These strategies must be enabled and reinforced by policy-level change such as sustainable funding arrangements. Ecological approaches that address social and environmental conditions have likewise been indicated for other Australian Aboriginal populations [23,24].

Knowledge translation activities have already commenced utilising these findings. The identified barriers to cessation have been presented back to stakeholders to facilitate brainstorming of stakeholder-derived strategies to combat the challenges of quitting. A consultative ground-up approach to action planning is considered paramount to the development of culturally relevant programs that can respectfully enable AHWs to quit smoking and remain abstinent.

Our findings are potentially transferable to AHWs and health professionals in a similar role in other regions across Australia and may also be relevant to Indigenous populations in other countries sharing a similar history of colonization and tobacco use. The sampling framework we employed (that included health service management and tobacco control personnel in addition to individual AHWs) was a strength of the study as it enabled the identification of barriers to quitting faced by AHWs in the workplace, community and broader policy level contexts that have not been previously elaborated. A lack of descriptive data for participants (e.g. age, qualifications) is a limitation of the study. The non-Aboriginality of the interviewers is another potential limitation of the data collection process because Aboriginal health staff may have felt uncomfortable sharing their stories with interviewers from a different background. Efforts were made to recruit Aboriginal interviewers but were unsuccessful. Hence, cultural mentorship was provided to non-Aboriginal interviewers by two Aboriginal research partners to ensure culture cultural protocols were respected and researchers felt comfortable conducting interviews. The cultural mentors assisted the development of interview questions and provided ongoing advice, support and feedback to interviewers during formal meetings and through informal post-interview debriefings. The interviewees who agreed to participate appeared comfortable with non-Aboriginal interviewers and shared intimate details of their experiences. This may reflect the successful cultural mentorship provided and the manner in which the Tackling Tobacco Coordinator vouched for the sincerity and credibility of the research team. The partnership and support of the Aboriginal Health Council of South Australia may have also enhanced the trust of health service managers and staff. Despite this, it is probable that smokers who were resistant to quitting would have been less likely to participate in our study. We collected shadowed data (where respondents shared stories about the experiences of others) [34], and invited Aboriginal stakeholders to contribute to the interpretation of findings to compensate for any potential sampling bias.

## Conclusions

There were multilevel factors that created barriers to quitting smoking in AHWs. Personal obstacles such as stress and grief, lack of knowledge about quit methods and lack of access to quit tools were reported. Peer influences were important and included a social pressure to smoke, social exclusion during quitting and lack of role models. In the workplace there were many AHWs who smoked, organisational practices that enabled smoking and challenges to implementation of Smokefree policy. A culture of smoking at the community level made it difficult for AHWs to avoid smoking. Participants also identified policy-level features such as inadequate funding of tobacco programs and lack of Smokefree public spaces as barriers to quitting. Considered together, an overall normalisation and tolerance of smoking existed in the social networks of AHWs which undermined their ability to quit.

These findings indicate that multidimensional smoking cessation programs are needed to support AHWs to quit smoking. For some AHWs, individual level features were predominant barriers, and for others social influences and pro-smoking environments were greater obstacles. Hence, flexible smoking cessation programs that can be targeted to local needs are indicated. Efforts aimed at enabling smoking reduction in AHWs need to challenge the normalisation of smoking in Aboriginal society. Offering AHWs culturally-relevant information, skills and resources to quit smoking is important, however success is more likely to occur if quitting is enabled through ecological programs that reduce the broader social and environmental stressors that cause burden in the lives of AHWs.

## Competing interests

The authors declared no potential conflicts of interest with respect to the research, authorship, and/or publication of this article.

## Authors’ contributions

AD undertook data collection and analysis and drafted the manuscript. HS provided cultural mentoring during the study, assisted in analysis and refined the manuscript. AC participated in the design and coordination of the study, provided cultural mentoring, assisted in analysis and refined the manuscript. MC and MD conceived of the study, were responsible for its design and coordination, and refined the manuscript. MC also undertook data collection and secondary review of the analysis. All authors read and approved the final manuscript.
